# Circulating immune cells and apolipoprotein A mediation: a Mendelian randomization study on hypertensive disorder of pregnancy

**DOI:** 10.3389/fimmu.2024.1438680

**Published:** 2024-09-17

**Authors:** Jingting Liu, Yawei Zhou, Yijun Dong, Wendi Wang, Yan Li, Jianying Pei

**Affiliations:** ^1^ Maternal and Child Health Care Research Center, Gansu Provincial Maternity and Child Care Hospital, Lanzhou, China; ^2^ The Second Hospital & Clinical Medical School, Lanzhou University, Lanzhou, China; ^3^ Department of Biochemistry and Molecular Biology, Medical College of Northwest Minzu University, Lanzhou, China

**Keywords:** hypertensive disorders of pregnancy (HDP), immunophenotype, Mendelian randomization, causality, mediation analysis

## Abstract

**Background:**

Studies using observational epidemiology have indicated that inflammation and immunological dysregulation are important contributors to placental and renal failure, which ultimately results in maternal hypertension. The potential causal relationships between the immunophenotypes and hypertensive disorder of pregnancy (HDP) are yet unclear.

**Methods:**

We conducted two-sample Mendelian randomization (MR) analyses to thoroughly examine the relationship between immunophenotypes and HDP. The GWAS data on immunological traits was taken from public catalog for 731 immunophenotypes and the summarized GWAS data in 4 types of HDP were retrieved from FinnGen database. The link between immune cell traits and HDP was examined through our study methodology, taking into account both direct relationships and mediation effects of apolipoprotein A (apoA). The inverse variance weighted (IVW) method served as the main analysis, while sensitivity analysis was carried out as a supplement.

**Results:**

We identified 14 highly correlative immunophenotypes and 104 suggestive possible factors after investigating genetically predicted immunophenotype biomarkers. According to the IVW analysis, there was a strong correlation between HDP and HLA DR on DC and plasmacytoid DC. Reverse MR analysis showed that there was no statistically significant effect of HDP on immune cells in our investigation. Mediation analysis confirmed that apoA mediates the interaction between HLA DR on DC and HDP.

**Conclusion:**

Our results highlight the complex interplay of immunophenotypes, apoA, and HDP. Moreover, the pathophysiological link between HLA DR on DC and HDP was mediated by the level of apoA.

## 1 Introduction

Hypertensive disorder of pregnancy (HDP), including chronic hypertension, gestational hypertension and pre-eclampsia/eclampsia, is one of the most common complications during pregnancy, affecting 5% to 10% of pregnancies (1). HDP continues to be one of the leading causes of maternal mortality globally (2, 3), which has a significant negative impact on the short- and long-term health of expectant mothers and their children (4, 5). Evidence now available indicates that maternal HDP may raise the possibility of high blood pressure, stroke, and cardiovascular diseases (6, 7). Apart from unfavorable consequences during pregnancy, HDP has also been linked to a higher likelihood of unfavorable birth outcomes for infants, including premature birth, low birth weight, and neonatal mortality (8). Although the exact mechanisms of HDP development are not fully understood, studies have suggested that the precise coordination between immune cells and trophoblasts plays a crucial role. The process of placentation and the formation of the maternal-fetal interface is intricate and requires meticulous coordination between immune cells and trophoblasts. Angiogenesis, inadequate trophoblast invasion, inefficient spiral uterine artery remodeling, and endothelial dysfunction are important processes behind the development of HDP (9, 10). Notably, immunological dysregulation had a significant role in the placental and renal dysfunction, and then lead to maternal hypertension (11). NK cells are the most prevalent leukocyte type in the decidua, which control spiral artery remodeling and trophoblast invasion (12, 13). They are drawn to the area by a variety of substances secreted by placental trophoblasts and decidual stromal cells. Uterine IL-15 release encourages dNK maturation. In turn, the mature dendritic NK cell secretes cytokines, such as TNFα, VEGF, and IFNγ, to facilitate blastocyst implantation and decidual remodeling (14, 15). Insufficient or inadequate regulation of the immune system, activation of innate immune cells and imbalanced differentiation of T helper (Th) cell subsets create a cytotoxic environment that results in oxidative stress, endothelial dysfunction and intrauterine growth restriction (16). Decidual CD4^+^CD25^+^Foxp3^+^Tregs subsets have been proposed to specifically contribute to pregnancy through reducing inflammatory responses and mediating immunological tolerance to fetal antigens (17). Increased CD4^+^CD25^high^Foxp3^+^ cell counts and decreased counts and functional activity of CD4^+^CD25^+^Foxp3high^+^ cell are linked to preeclampsia, compared to a normal pregnancy (18).

Disturbances in maternal lipid metabolism has been reported to be a risk factor for developing pregnancy complications and adverse perinatal outcomes. A population-based prospective study reported that the high level of triglycerides (TG) increased risk of gestational hypertension (OR: 1.81, 95% CI: 1.16–2.82) and preeclampsia (OR: 1.63, 95% CI: 1.19–2.25) (19). And the result from a large cohort study with 8407 primigravid women indicated that elevated TG and decreased high density lipoprotein (HDL) cholesterol were associated with increased odds of pre-eclampsia (20). Several studies reported the level of plasma lipoprotein (a) in women with preeclampsia and normotensive pregnant women. The plasma lipoprotein(a) levels were increased in pregnant women with preeclampsia compared with normal pregnancy (21, 22). However, Djurovic S, et al. mentioned that the decreased lipoprotein (a) levels were linked to in preeclampsia, and they didn’t find any significant association between lipoprotein(a) level and the severity or complications of preeclampsia (23). In addition, Leerink CB, et al. reported that there were no statistically significant difference of plasma lipoprotein(a) levels existed between normotensive pregnant and pre-eclamptic women (24). The study design, race of the population, sample size, the laboratory determination of lipoprotein(a) varied significantly among published literatures, there is no clear consensus on the significance of lipoprotein(a) in normal and complicated pregnancy (25). A retrospective cohort study showed that Apo-A levels of pregnant woman with preeclampsia (1.53 ± 0.35) was lower when compared with those with chronic hypertension (P = 0.004) at 4-16 weeks of pregnancy, the situation was similar at 28-42 weeks(preeclampsia:1.94 ± 0.34 vs gestational hypertension: 2.00 ± 0.34, P = 0.002) (26). According to a study by Serrano NC et al., pre-eclampsia was also found to be negatively correlated with apoA1 and positively linked to apoE and the apoB/apoA1 ratio (20). However, a previous study conducted in1997 showed that the presence of apo(a) isoforms are not risk factors for the development of preeclampsia (27). Nevertheless, the majority of the previously cited findings came from observational or retrospective research, which may have been limited by the diverse patient organization and small sample size. They had only identified correlations between different immune cells, maternal lipid levels and hypertensive disorders in pregnancy; whether these correlations are causal remains to be determined. The restricted sample size and confounding factors may cause bias in the results.

Mendelian randomization (MR) analysis, a statistical method that uses genetic variants as instrumental variables (IVs) to examine potential causal relationships between exposures and outcomes, has become widely used (28, 29). Without using randomized controlled trials or animal research, it is a very effective method for exploring causation from observed associations (30). Compared with observational study, MR analysis has two advantages: since genetic differences are randomly dispersed during meiosis, there is little chance that they will correlate with environmental factors (31); the distribution of genotypes precedes the time of acquired exposure, and reverse causality has little effect on causal estimation (32). MR has gained popularity for evaluating possible causal links between risk factors and HDP due to its time- and cost-efficient benefits. The role of exposure factors, including cardiovascular disease-related proteins (33), circulating adipokine levels (34), and gut microbiota (35), has been demonstrated in HDP by previous studies. Hosier H et al. mentioned that elevating HDL-C was linked to a lower incidence of preeclampsia (OR= 0.84, P=0.004, 95%CI: 0.74-0.94) (36). But a study conducted by M Golawski et al. showed that lipoprotein(a) levels do not appear to have a causal role in preeclampsia (37). Nevertheless, whether immune cells could affect the progression of HDP mediated by lipid or apolipoproteins is still uncertain. In order to fill the aforementioned vacuum, a comprehensive MR analysis involving 731 inflammatory cells, plasma apolipoproteins, and HDP was conducted. We additionally inquired into the pathways that plasma apolipoprotein A to mediate the relationship between peripheral immune cells and HDP.

## 2 Materials and methods

### 2.1 Study design

**Figure 1:**
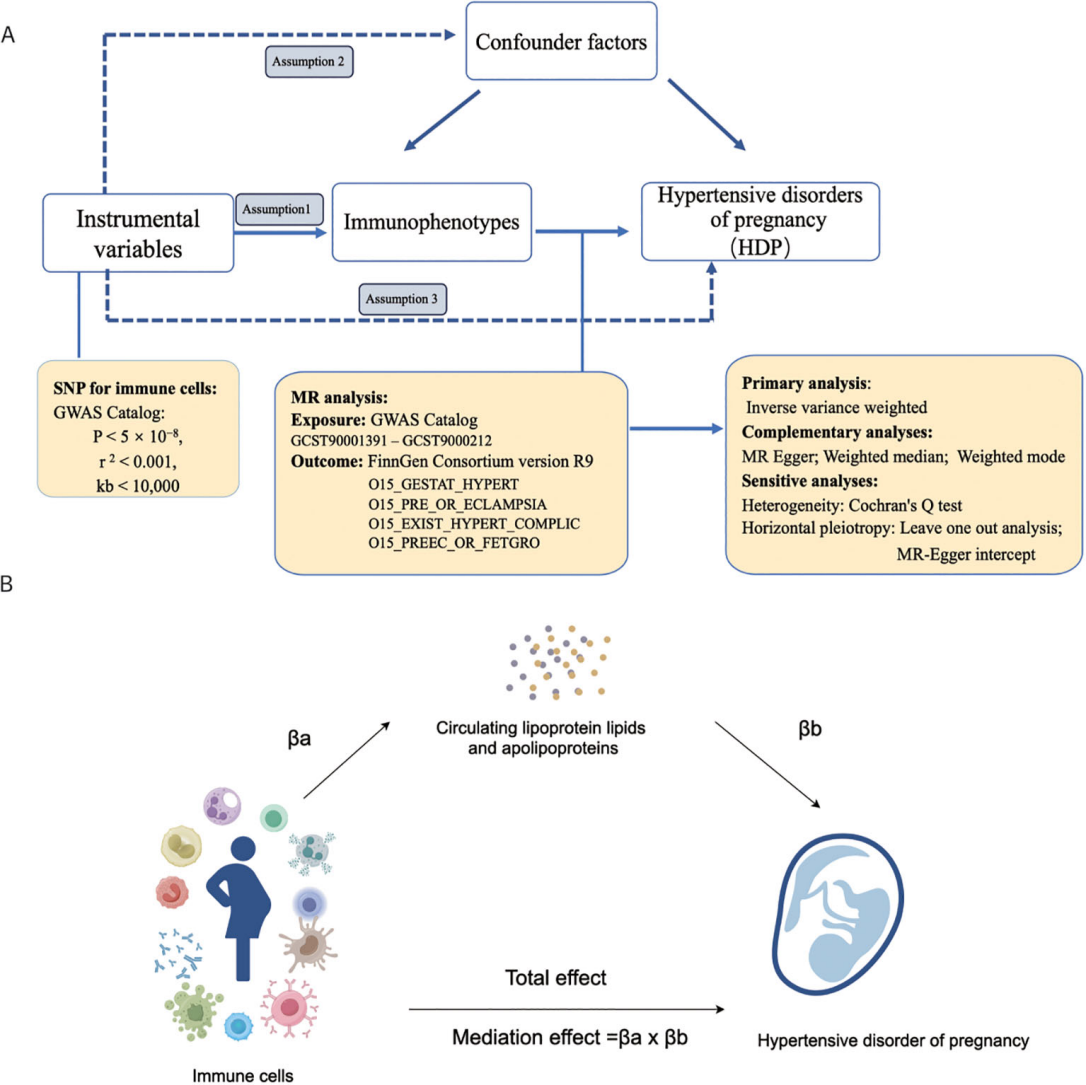
The workflow of this study design. **(A)** Two-sample Mendelian randomization analyses between 731 immunophenotypes and hypertensive disorders of pregnancy; **(B)** Mediation analysis in this study. GWAS, the genome wide association studies; SNPs, single-nucleotide polymorphisms; IVs, instrumental variables; HDP, hypertensive disorders of pregnancy; EH, pre-existing hypertension complicating pregnancy, childbirth and the puerperium; GH, gestational hypertension; PE, pre-eclampsia or eclampsia; PF, pre-eclampsia or poor fetal growth; MR, Mendelian randomization.

**Figure 1** depicts the flowchart used in the current study. Using MR analysis and mediation analysis, we assessed the causative relationship between 731 immunophenotypes and HDP. We also looked into the possibility that plasma apolipoproteins could operate as a mediating factor in this causal relationship. First, we collected the 731 immunophenotypes, plasma apolipoproteins, and HDP from the publicly available GWAS summary data. Three basic assumptions must hold while choosing IVs (**Figure 1A**): (1) The IVs must have a strong connection with the exposure; (2) The IVs not be linked to any other confounders; (3) IVs merely affect outcome through exposure and ignoring any other pathways (38, 39). In addition, we employed two sample MR analyses to assess the causal association between HDP and peripheral immune cells. We additionally discovered that HDP and plasma apolipoproteins were related. Finally, we performed a mediation analysis to quantify the proportion of the total effect of highly correlative immunophenotypes on the risk of HDP that was mediated by plasma apolipoproteins.

### 2.2 Exposure and outcome data sources

The 731 immunophenotypes of peripheral blood are accessible through the GWAS Catalog (GCST90001391-GCST90002121) retrieved from the study conducted by Valeria Orrù et al. (40). The original GWAS on immunophenotypes was performed using data from 3,757 European individuals. It contains 4 trait types,7 panels, and 731 traits. When immunophenotypes traits were classified in 7 subtypes (B cell, cDC, maturation stages of T cell, monocyte, myeloid cell, TBNK, Treg). The characterization of immunophenotypes was presented in **Supplementary Table 1**. The Single Nucleotide Polymorphism (SNP) data of apolipoprotein A (apoA) levels (ID: ebi-a-GCST90013993) was obtained from the large-scale mapping of the genetics of the proteome conducted by Mbatchou J et al. with 355,859 white British individuals (41). The GWAS data of HDP were obtained from the FinnGen database (https://www.finngen.fi/en) R9 version. In accordance with the HDP definition, we included 4 cohorts. The 4 cohorts applied in this study were gestational hypertension (GH), pre-eclampsia/eclampsia (PE), pre-eclampsia or poor fetal growth (PF), and pre-existing hypertension (EH). The detailed information of datasets used in this study was displayed in **Table 1**. All of the relevant data were collected from pertinent literature and publicly accessible databases, based to participant agreement and ethical approval. This means that there is no need for the ethical approval from the institutional review board.

**Table 1:** The GWAS datasets used for analyses.

Trait/Disease	Data Type	Consortium	GWAS_ID	Sample Size	Case	Control
Immunophenotypes	Exposure	GWAS Catalog	GCST90001391 - GCST9000212	3757	-	-
Gestational hypertension	Outcome	Finngen	O15_GESTAT_HYPERT	202768	8502	194266
Pre-eclampsia or eclampsia	Outcome	Finngen	O15_PRE_OR_ECLAMPSIA	201478	7212	194266
Pre-existing hypertension complicating pregnancy, childbirth and the puerperium	Outcome	Finngen	O15_EXIST_HYPERT_COMPLIC	196489	2223	194266
Pre-eclampsia or poor fetal growth	Outcome	Finngen	O15_PREEC_OR_FETGRO	210870	10297	200573
Apolipoprotein A (ApoA) levels	Mediator	UK Biobank	ebi-a-GCST90013993	355859	–	–

### 2.3 Instrumental variable selection

To guarantee the causal relationship between immunophenotypes, apoa level and HDP was accurate and reliable, we involved a series of quality tests to select IVs that satisfied the three assumptions of MR analysis. The procedures we used to choose IVs were as follows:

Firstly, we applied a strict significance threshold to filter out SNP that were closely associated with immunophenotypes and HDP. The criteria for the genetic instruments were as follows: P < 5 × 10^−8^. Meanwhile, since the existence of linkage disequilibrium (LD) would lead to bias, we set the LD of SNPs which met “r^2^ < 0.001 and kb > 10,000” to guarantee the independence of the aggregated SNPs. Moreover, to avoid weak instrumental bias, we evaluated the strength of the IV correlations by calculating the F-statistic and selected SNPs with F-statistic >10 for inclusion in this MR analysis (MR Assumptions1). Secondly, the SNPs that showed a significant association with HDP (P < 1 × 10^−5^) were eliminate (MR Assumptions2). Thirdly, we looked through and eliminated SNPs corresponding to confounders via the PhenoScanner website (MR Assumptions3) (42). Finally, 2626 independent SNPs were obtained as IVs for immunophenotypes.

When apoA level was used as exposure, considering the limited number of available SNPs, we selected SNPs with a loose cutoff of P < 5 × 10^−5^, and its criteria were as follows: P < 5 × 10^−5^, r ^2^ < 0.001, and kb < 10,000. And 297 independent SNPs were obtained for apoA level.

### 2.4 Statistical analysis

We utilized 4 MR methods, including inverse variance weighted (IVW) (43), MR Egger (44), Weighted median (45), Weighted mode, to determine the causal link between immunophenotypes and HDP. Considering IVW often offers the most statistical power, it serves as the primary analysis. The effect was represented by the odds ratio (OR) and 95% confidence interval (CI). The sensitivity analyses were performed to evaluate the robustness of the findings, including heterogeneity and pleiotropy. The heterogeneity of the chosen IVs was investigated using Cochran’s Q statistic and the corresponding P-values, and P >0.05 was considered to indicate no heterogeneity (46). To identify horizontal pleiotropic effects, the MR-Egger regression intercept test was performed (44), and leave-one-out sensitivity analysis was used to assess whether a single SNP drove the causal estimation. To explore reverse causality, we applied the MR Steiger directionality test (47). Moreover, considering the issue of multiple testing, false discovery rate (FDR) correction was performed. Immunophenotypes with adj.P value <0.05 and have causal links with 4 cohorts of HDP were thought to have highly correlative factors, whereas those showed a P value <0.05 but 0.05< adj.P value <0.2 were considered suggestive of a causal relationship. All analyses were carried out in R software 4.3.1 utilizing the “Two Sample MR” (48) and “Mendelian Randomization” packages.

### 2.5 Mediation analysis

The objective of mediation analysis is to explain the potential mechanisms by which an exposure influences an outcome. Traditional methods of mediation analysis encounter several methodological challenges because of confounding between an exposure, mediator, and measurement error. But mediation analysis can benefit from using MR to strengthen causal inference (49). In order to explore potential mediating pathways of plasma apolipoproteins and immune cells in the causal link to HDP, we conducted a mediation analysis. Because HLA DR on myeloid Dendritic Cell and HLA DR on Dendritic Cell were both highly correlative factors and they had causative effects on 4 cohorts of HDP (GH, PE, PF and EH), we plan to further investigate the causative relationship and potential mediating pathways between dendritic cell and HDP.

Firstly, we used two-sample MR analysis to assess whether immunophenotypes and apoA level are causally related. This step was analogous to our preliminary analysis, and the causative effect we obtained in this step is βa. Secondly, multivariable MR (MVMR) was employed to determine the causative relationship between apoA and HDP. In this step, inverse variance weighted (multiplicative random effects) was the main analysis method to ensure that the mediating effects on outcomes are independent of exposure, and the causative effect we obtained in this step is βb. Thirdly, we calculated the mediation effect of the dendritic cell on HDP through apoA level by multiplying together the estimates from steps above. We also calculated the weight of apoA by dividing the mediated effect by the total effect (the beta estimate in two-sample MR analysis between immunophenotypes and HDP). The formula of mediating effect was as follow: Mediation effect = βa × βb; Direct effect = total effect - mediation effect; Mediation Proportion= mediation effect/total effect×100%.

## 3 Results

### 3.1 Causal effects of immunophenotypes on HDP

**Figure 2:**
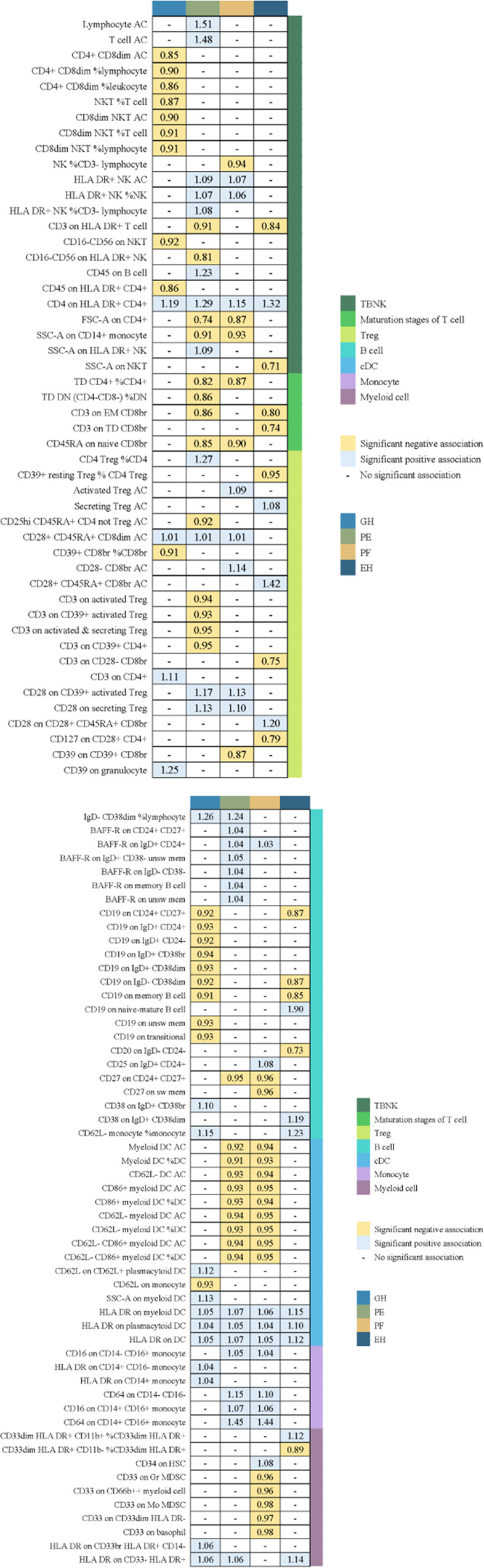
Summary of associations of genetically predicted immunophenotype traits with hypertensive disorders of pregnancy. EH, pre-existing hypertension complicating pregnancy, childbirth and the puerperium; GH, gestational hypertension; PE, pre-eclampsia or eclampsia; PF, pre-eclampsia or poor fetal growth.

**Figure 3:**
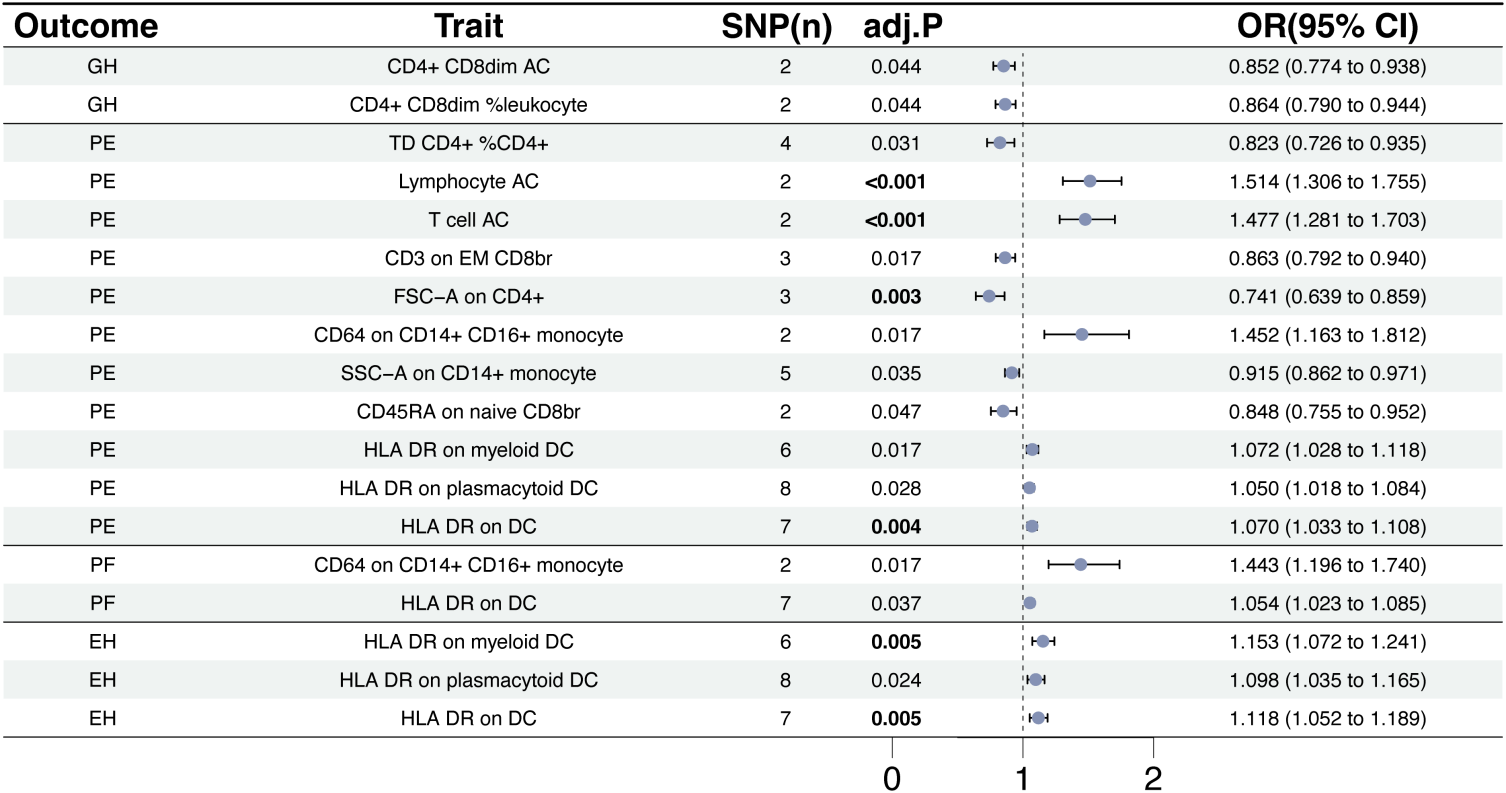
The causal effect of immunophenotypes on hypertensive disorders of pregnancy. EH, pre-existing hypertension complicating pregnancy, childbirth and the puerperium; GH, gestational hypertension; PE, pre-eclampsia or eclampsia; PF, pre-eclampsia or poor fetal growth; SNP(n), the number of single-nucleotide polymorphisms; OR, odds ratio; CI, confidence interval.

We used a tow-sample MR analysis to get insight into the connection between immunophenotypes and 4 types of HDP. The 4 cohorts applied in this study were GH, PE, PF, and EH. After the selection of IVs, 2626 independent SNPs associated with immunophenotypes were found in this study. 14(18 pairs) highly correlative immunophenotype traits (adj.P value <0.05) and 104(150 pairings) suggestive possible factors (P value <0.05, 0.05< adj.P value <0.2) had causal effects on HDP. The results of MR analysis between 4 HDP types and immune cells are presented in **Figures 2**, **3** and **Supplementary Table 3**. When GH was used as outcome, MR analysis was conducted on 731 immunophenotypes with adj.P value <0.05 as criteria, only 2 highly correlative factors, including CD4^+^CD8^dim^ AC (OR= 0.852, P= 1.16E-03, 95%CI: 0.774 - 0.938) and CD4^+^CD8^dim^ %leukocyte (OR= 0.864, P= 1.27E-03, 95%CI: 0.79 - 0.944), were linked to a reduced risk of GH. When adj.P value <0.2, we noticed associations between 36 immune cells(suggestive possible factors) and the risk of GH. Among them, the B-cell panel showed the highest number of significant associations when compared to other panels, and the TBNK panel went the second (**Figure 2**, **Supplementary Table 3**). As depicted in **Figure 2**, 11 highly correlative immunophenotypes (**Figure 3**) and 50 suggestive possible factors (**Supplementary Table 3**) had causal effects on PE. HLA DR on myeloid DC (OR= 1.072, P= 1.09E-03, 95%CI: 1.028 - 1.118), HLA DR on plasmacytoid DC (OR= 1.05, P= 2.18E-03, 95%CI: 1.018 - 1.084), HLA DR on DC (OR= 1.07, P= 1.36E-04, 95%CI: 1.033 - 1.108), Lymphocyte AC (OR= 1.514, P= 3.84E-08, 95%CI: 1.306 - 1.755), T cell AC (OR= 1.477, P= 8.30E-08, 95%CI: 1.281 - 1.703), and CD64 on CD14^+^ CD16^+^ monocyte (OR= 1.452, P= 9.71E-04, 95%CI: 1.163 - 1.812) were associated with an elevated risk of PE; while TD CD4^+^ %CD4^+^ (OR= 0.823, P= 2.62E-03, 95%CI: 0.726 - 0.935), CD3 on EM CD8^br^ (OR= 0.863, P= 7.56E-04, 95%CI: 0.792 - 0.94), FSC-A on CD4^+^ (OR= 0.741, P= 7.08E-05, 95%CI: 0.639 - 0.859), SSC-A on CD14^+^ monocyte (OR= 0.915, P= 3.41E-03, 95%CI: 0.862 - 0.971), and CD45RA on naive CD8^br^ (OR= 0.848, P= 5.17E-03, 95%CI: 0.755 - 0.952) played inhibitory roles. To deepen our understanding of the potential associations between the immune cells and HDP, we expanded our analysis to PF and EH cohort. CD64 on CD14^+^ CD16^+^ monocyte and HLA DR on DC had a positive association with PF. In addition, 3 highly correlative facilitators (HLA DR on myeloid DC, HLA DR on plasmacytoid DC, and HLA DR on DC) were linked to an increased risk of EH. For illustrating the trends in immunophenotypes under the four MR methods, scatter plots were summarized in **Supplementary Figure 1**.

### 3.2 Sensitivity analysis

Sensitivity analyses were conducted to investigate the pleiotropy and heterogeneity of exposure to outcome in order to evaluate the robustness of the validity of the causal evaluation. The MR-Egger intercept test and Cochran’s Q test revealed no pleiotropy or heterogeneity (**Supplementary Table 4**), demonstrating the robustness of our results. These results were corroborated by the funnel plots (**Supplementary Figure 3**). None of the SNPs was able to independently change the causal estimate, according to leave-one-out analysis (**Supplementary Figure 4**). Subsequently, the MR Steiger directionality test was conducted, showing no evidence of reverse causality (**Supplementary Table 5**).

### 3.3 Causal effects of immunophenotypes on HDP by cardiovascular protein/Mediation analysis results

Because HLA DR on DC and HLA DR on myeloid DC were highly correlative traits on the risk of HDP, and they were also causally associated with the 4 HDP cohorts used in this study. Mediation analysis of apoA was conducted to explore whether the effect of DCs on HDP was mediated by it. Firstly, we evaluated the causal links between immunophenotypes and apoA level. In IVW method, we only found that HLA DR on DC (OR= 1.022, P=2.22E-03, 95%CI: 1.008- 1.038) and HLA DR on myeloid DC (OR= 1.022, P=3,00E-05, 95%CI: 1.012-1.033) had a positive association with apoA levels; while the apo level played inhibitory roles in GH(OR=0.88), PE(OR=0.84), PF(OR=0.91), and EH(OR=0.77). **Supplementary Table 6** provides comprehensive details about the random effects of MR analysis between immunophenotypes, apoA and HDP. No evidence of heterogeneity was found using the Cochran’s Q statistic test (**Supplementary Table 7**). According to the result of MR-Egger intercept method (**Supplementary Table 8**) and leave-one-out sensitivity analysis (**Supplementary Table 9**), there was no evidence of horizontal pleiotropy.

**Figure 4:**
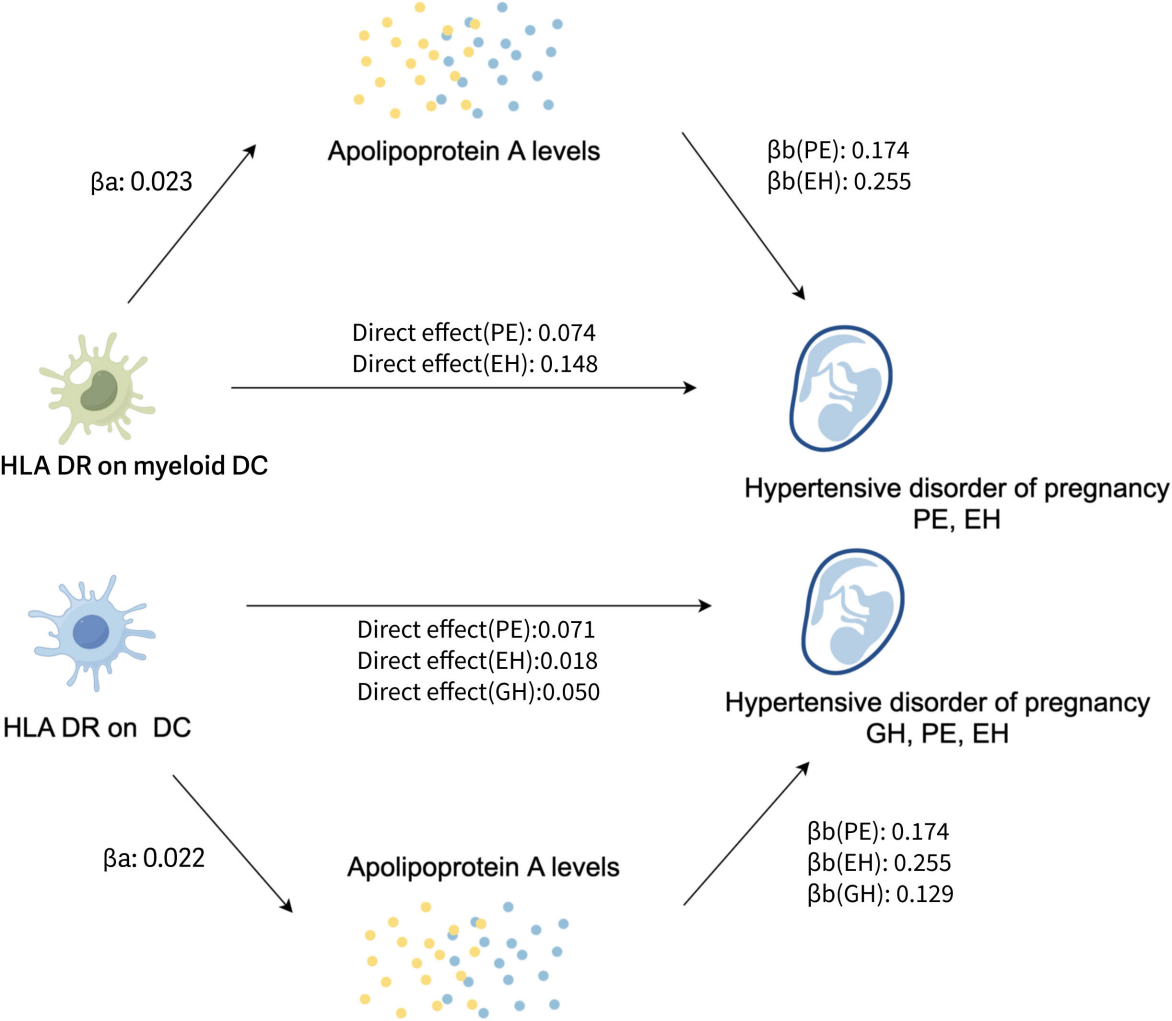
The potential pathogenesis of HDP in this study: the proposed causal interactions of DC and apoA level on HDP (Created with Figdraw). βa, causal effect of DCs on apoA levels; βb, causal effect of apoA levels on HDP; Direct effect, the effect of the exposure (DCs) on the outcome (the risk of HDP) through the candidate mediator (apoA levels); EH, pre-existing hypertension complicating pregnancy, childbirth and the puerperium; GH, gestational hypertension; PE, pre-eclampsia or eclampsia; DC, dendritic cell; aopA, apolipoprotein A.

To investigate whether apoA level mediation mediated the effect of DCs on HDP, mediation analysis was performed. The mediation effect of DCs on HDP via apoA level was presented in **Table 2**. There was evidence for a mediating role of apoA level (-5.64% mediated, P =0.029) in the relationship between HLA DR on myeloid Dendritic Cell and PE. And we also found that apoA had a mediating role in the causality between HLA DR on myeloid Dendritic Cell and EH (-4.02%, P= 0.042). The similar mediating effort was found in the causal effort on Dendritic Cell in GH (-6.04% mediated, P =0.042), PE (-5.72% mediated, P =0.013), and EH (-5.04% mediated, P =0.023). The results suggested that apoA may act as a mediator of the causal effect between DCs and HDP (**Figure 4**).

**Table 2:** Mediation effect of immunophenotypes on via Apolipoprotein A levels.

Exposure	Mediator	Outcome	Total effect ^a^	Direct effect ^b^	Mediation effect (95% CI)	P-value	Mediation Proportion (95% CI)
HLA DR on myeloid Dendritic Cell	Apolipoprotein A levels	GH	0.050	0.053	-0.003 (-0.006 ~ 2.74E-04)	0.064	-5.79%(-12.13%-0.55%)
HLA DR on myeloid Dendritic Cell	Apolipoprotein A levels	PE	0.070	0.074	-0.004 (-0.008 ~ -3.08E-04)	**0.029**	-5.64%(-10.84%~ -0.44%)
HLA DR on myeloid Dendritic Cell	Apolipoprotein A levels	EH	0.143	0.148	-0.006 (-0.011 ~ -3.60E-05)	**0.042**	-4.02%(-8.01%~ -0.23%)
HLA DR on Dendritic Cell	Apolipoprotein A levels	GH	0.047	0.050	-0.003 (-0.006 ~ -4.00E-05)	**0.042**	-6.04%(-12.01%~ -0.08%)
HLA DR on Dendritic Cell	Apolipoprotein A levels	PE	0.068	0.071	-0.004 (-0.007 ~ -0.001)	**0.013**	-5.72%(-10.29%~ -1.14%)
HLA DR on Dendritic Cell	Apolipoprotein A levels	EH	0.112	-0.006	-0.006 (-0.011 ~ -0.001)	**0.023**	-5.04%(-9.47%~ -0.61%)

When the 95% CI of the mediation effect spans 0, the 95% CI for mediation proportion is not calculated, as the direction of the upper or lower limit of the mediation effect is opposite to the total effect; Bold formatting indicates that the P-value is less than 0.05; CI, confidence interval.

a Total effect is defined as the causal effect of the exposure (immune cells-DCs) on the outcome (the risk of HDP).

b Direct effect is defined as the effect of the exposure (immune cells-DCs) on the outcome (the risk of HDP) through the candidate mediator (apolipoprotein A levels).

## 4 Discussion

**Figure 5:**
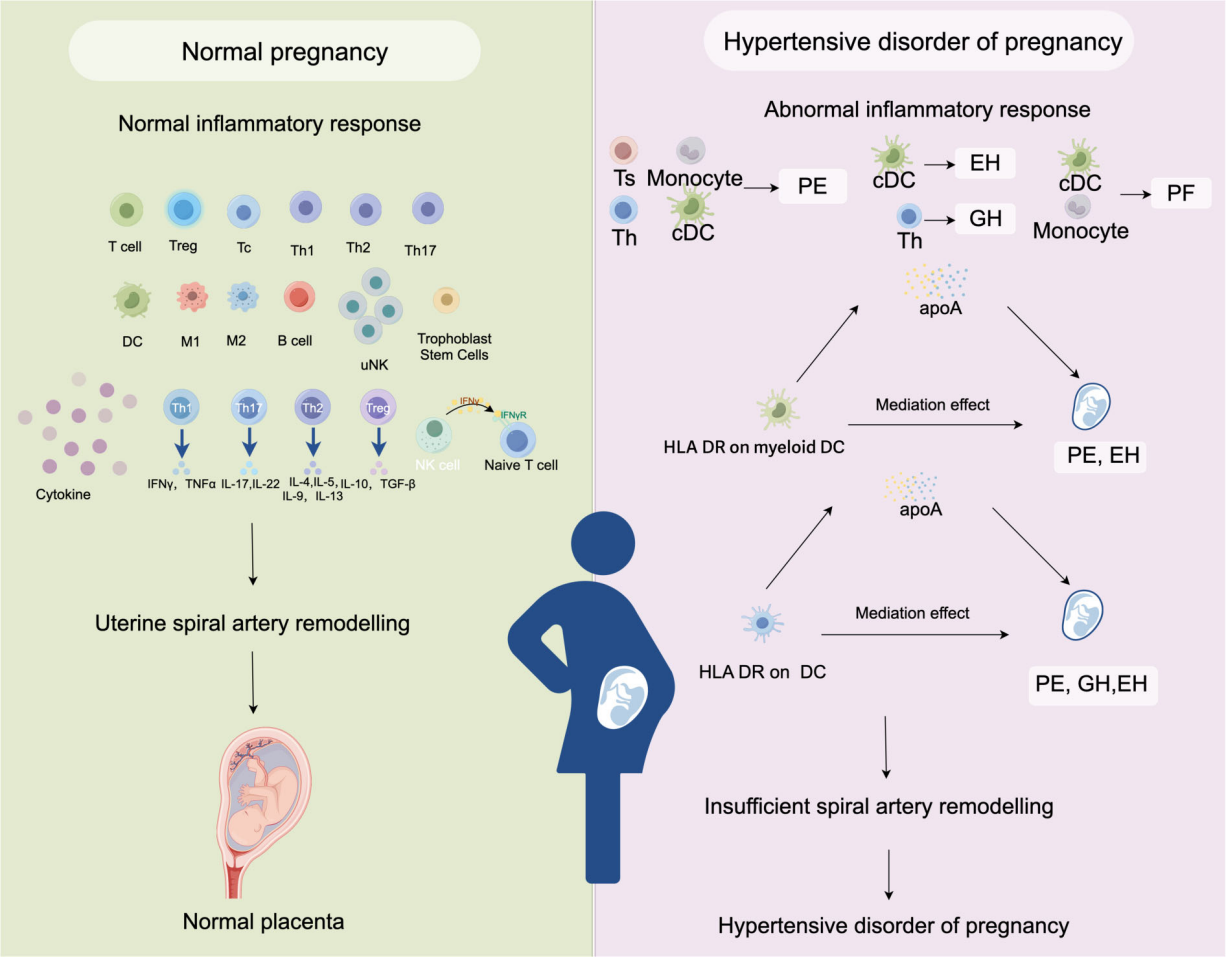
A schematic summary figure for the positive results after the Bonferroni method. (Created with Figdraw). EH, pre-existing hypertension complicating pregnancy, childbirth and the puerperium; GH, gestational hypertension; PE, pre-eclampsia or eclampsia; PF, pre-eclampsia or poor fetal growth.

HDP is a major contributor to maternal mortality and morbidity. In addition to jeopardizing maternal fertility, female patients will experience psychological distress. The pathogenesis and pathophysiology of HDP are very complex and multifactorial. The occurrence of preeclampsia is mainly related to the following factors: defective remodeling of spiral vessels, cytokine imbalance, endothelial dysfunction, oxidative stress, and so on (50). Numerous research conducted recently have linked HDP to immunological imbalance. Despite the rich results of previous observational studies, it is difficult to identify the causes of changes in inflammatory cells in patients with HDP through general observational studies. In this study, we used an MR analysis to explore the potential causal effects between inflammatory cells and HDP. We analyzed the relationships between 731 immunophenotypes and 4 types of HDP (GH, PE, PF, and EH). The results showed that some immune cells were highly correlative factors of HDP. The cDC panel had 35 pairs immunophenotype of significant associations, which is the largest number than other panels. We found that the median fluorescence intensity (MFI) of HLA DR on DC increased the risk of PE by 7.0% (OR=1.070, adj.P= 0.004), the risk of PF by 5.4% (OR=1.054, adj.P= 0.037),and the risk of EH by 11.8% (OR=1.118, adj.P= 0.005). Similarly, the MFI of HLA DR on myeloid DC increased the risk of PE by 7.2% (OR=1.072, adj.P= 0.017) and the risk of EH by 15.3% (OR=1.153, adj.P= 0.005). Importantly, our findings suggest that apoA acts as a mediator of the impact of DC on HDP. We generated a schematic summary figure in **Figure 5** to aid in public understanding of the outcomes of our study. Overall, the study provided more evidence for the link between immunophenotype and HDP than we had anticipated.

The immune system is balanced during a healthy pregnancy to ensure a successful implantation while shielding the embryo from immunological assaults (51). In our study, we found that T cells (including CD4^+^ T cell, Terminally Differentiated CD4^+^ T cell, Effector Memory CD8^+^ T cell, and naive CD8^+^ T cell) were associated with a reduced risk of PE. Treg cells are essential for preserving the anti-inflammatory decidual milieu. They also govern the decidual leukocyte network, which promotes trophoblast invasion and cytotrophoblast growth, and hence regulate placental development and implantation (52). According to a recent research based on single-cell RNA sequencing, patients with preeclamptic showed a significant reduction in the percentage of monocytes (10.9% vs. 28.0%, P < 0.05) and an increase in the percentage of T cells (67.2% vs. 52.1%, P < 0.05) (53). Decidual macrophages produce angiogenic factor during pregnancy, which aids in the remodeling of spiral arteries (54). Throughout the first trimester (less than 12 weeks gestation), M1 macrophages (pro-inflammatory macrophages) dominate and play a role in embryo implantation, placental formation, and embryo growth. M2 macrophages (anti-inflammatory macrophages) prevail until labor when the placenta has fully grown in the second trimester (55). In contrast to a healthy pregnancy, preeclampsia is associated with a sustained increase in the M1-to-M2 ratio. Our results found that CD14^+^ CD16^+^ monocyte was an elevated risk factors of PE (OR= 1.452, P= 9.71E-04, 95%CI: 1.163 - 1.812). Castleman, J. S’s previous studies have revealed that monocyte count was significantly higher in pregnant women with previous hypertension compared to pregnant women without previous hypertension and non-pregnant controls (56), which supports our findings.

DCs are potent antigen-presenting cells that possess a special ability to carry out the vital function of triggering and adjusting immune responses (57). Unfortunately, the percentage of DCs in peripheral blood mononuclear cells is only 1.5%, and the process of enriching blood and tissue DCs is time-consuming (58). The investigation of DCs’ function in HDP met great challenges. According to our research, DC’s increased HLA-DR expression level may have a negative impact on HDP. Additionally, Li J. et al. discovered that pre-eclampsia patients had a peripheral blood myeloid DC to plasmacytoid DC ratio (1.86 ± 0.76) that was considerably greater than that of a normal pregnant woman (0.78 ± 0.41) (59). Immunohistochemistry analysis of preeclamptic decidua revealed the infiltration of both immature and mature dendritic cells (60). Another study found a potential correlation between increased Th1-type immunity and higher levels of CD1c^+^ in pre-eclamptic patients (61). Our results aligned with the previously cited research, indicating that DCs are associated with immunological tolerance and have a significant function in HDP.

Notably, far more pronounced alterations in lipid homeostasis are usually linked to preeclampsia. According to a meta-analysis, pregnant women who proceed on to develop preeclampsia have higher levels of maternal serum total cholesterol, non-HDL-C, and triglycerides than pregnant women who maintain normal blood pressure (62). ApoA-I is required for normal HDL maturation and metabolism (63).The human placenta is an endocrine secretory tissue that secretes apolipoproteins, specifically apoA1 and apoE. Melhem H et al. determined the directionality of apoA1 and apoE release by the human placenta for the first time, and they discovered that the placenta can secrete apoA1 and apoE to both the maternal and the fetal side (64). ApoA1 secretion in the human placenta perfusion model was primarily orientated towards the maternal side, and the increased levels of antiatherogenic apoA1 and apoE in the maternal blood may protect the mother from health complications arising from high lipid levels during pregnancy (64). But the role and importance of apoA in HDP have been seldom investigated. In our study, we identified that high level of apoA is a protective factor of 4 types HDP (GH, PE, PF and EH), the results of MR mediator analysis showed that apoA could mediated the MFI on DC, leading to HDP. The large cohort study conducted by Walldius, G. A er al. discovered that low levels of apoA-1 were associated with increased levels of apoB and apoA-1 is a protective factor of major adverse cardiovascular events (63). Apart from its significant anti-atherogenic function, the apoA-1 protein also contains protective cofactors with anti-inflammatory, anti-oxidative, and anti-thrombotic properties.

This is the first study to use Mendelian randomization method to examine the causal links between immunophenotypes and HDP mediated by cardiovascular proteins. Here are some benefits of our study: First of all, there is little chance that genetic variants during meiosis will link with environmental confounders because they are random. Secondly, the circulating immune cells and HDP data were taken from different samples, and two-sample data prevented bias from weak instrumental variables. Thirdly, compared to comparable observational studies, our methodology worked more statistically efficiently because of the high sample size and thorough coverage of immune cells. However, our study has several limitations. First off, bias may have resulted from the study’s stringent IVs filtering cut-off, which reduced the number of SNPs accessible for some immunophenotype traits. Secondly, Gender differences may affect MR results, because the SNP data of immunophenotype were obtained in both male and female populations, but HDP is exclusively found in female. Thirdly, given that all of the GWAS summary data were from populations in Europe, more investigation is needed to determine whether our findings apply to other racial or ethnic groups.

## 5 Conclusion

To sum up, our research exposed fascinating details on the relationship between immune cells and HDP. Crucially, it appears from our research that apoA mediates the effect of DC on HDP. Our research revealed distinct inflammatory cell characteristics for the disease’s onset and progression, providing new insights for HDP treatment, surveillance, and prevention.

## Data Availability

The original contributions presented in the study are included in the article/**Supplementary Material**. Further inquiries can be directed to the corresponding authors.
