# Supplementary material for: Circulating immune cells and apolipoprotein A mediation: a Mendelian randomization study on hypertensive disorder of pregnancy
Source: Front Immunol. 2024 Sep 17;15:1438680. doi: 10.3389/fimmu.2024.1438680 (PMC11442235; doi:10.3389/fimmu.2024.1438680)
Supplement: Supplementary file 1 [file DataSheet1.zip › supplementary Material/Supplementary Figure Legend-hdp.docx]

**Supplementary Figure Legend**

Figure S1. Scatter plot for the relationship between the SNP effect size of causal immune traits (x-axis) and the corresponding effect size estimates of HDP (y-axis).

A-C. pre-existing hypertension complicating pregnancy, childbirth and the puerperium; D-E. gestational hypertension; F-G. pre-eclampsia or poor fetal growth; H-R. pre-eclampsia or eclampsia.

Figure S2. Forest plot for the relationship between the immune traits and HDP by MR analysis. The significance of red lines are MR results of inverse variance weighted method and/or MR Egger test.

A-C. pre-existing hypertension complicating pregnancy, childbirth and the puerperium; D-E. gestational hypertension; F-G. pre-eclampsia or poor fetal growth; H-R. pre-eclampsia or eclampsia

Figure S3. Funnel plot of immune traits on HDP. The funnel plots are symmetric, which shows that the absence of heterogeneity.

A-C. pre-existing hypertension complicating pregnancy, childbirth and the puerperium; D-E. gestational hypertension; F-G. pre-eclampsia or poor fetal growth; H-R. pre-eclampsia or eclampsia

Figure S4 Leave-one-out analysis result of SNPs associated with dried fruit intake and risk on HDP.

A-C. pre-existing hypertension complicating pregnancy, childbirth and the puerperium; D. pre-eclampsia or poor fetal growth; E-L. pre-eclampsia or eclampsia
