# Supplementary figures and images for: Circulating immune cells and apolipoprotein A mediation: a Mendelian randomization study on hypertensive disorder of pregnancy
Source: Front Immunol. 2024 Sep 17;15:1438680. doi: 10.3389/fimmu.2024.1438680 (PMC11442235; doi:10.3389/fimmu.2024.1438680)

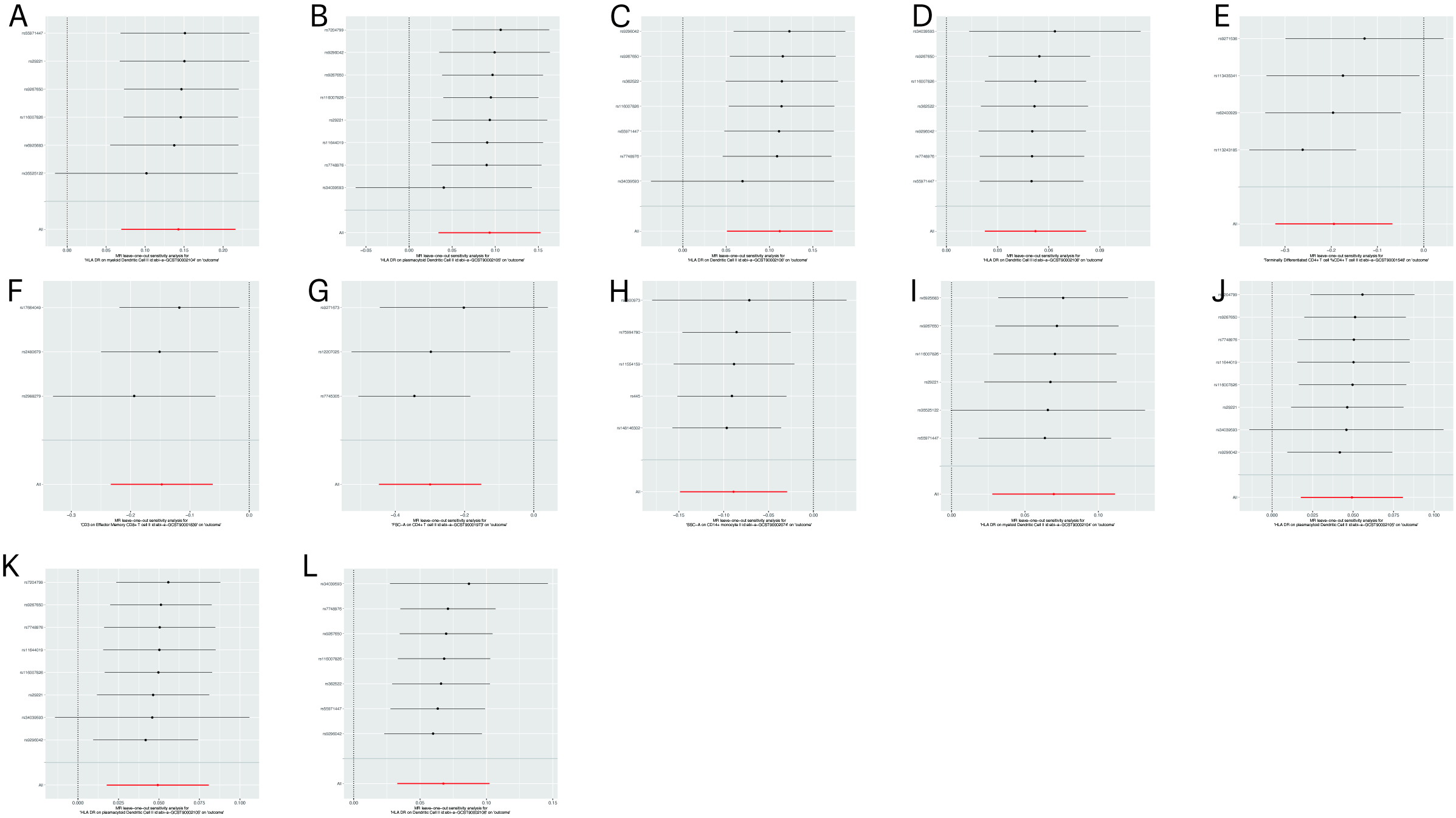

Supplement: Supplementary file 1 [file DataSheet1.zip › supplementary Material/FS4-HDP.tif]

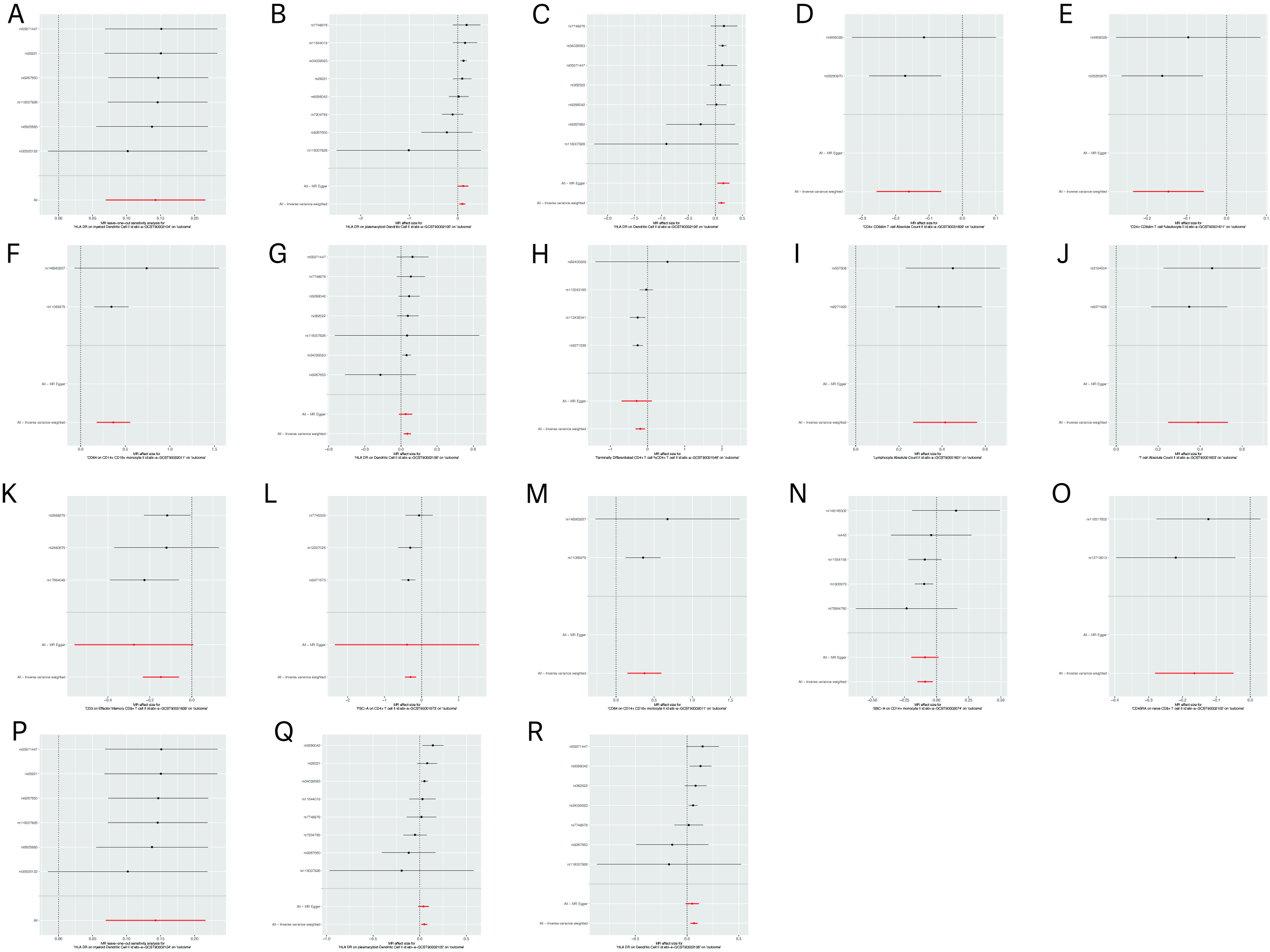

Supplement: Supplementary file 1 [file DataSheet1.zip › supplementary Material/FS2-HDP.tif]

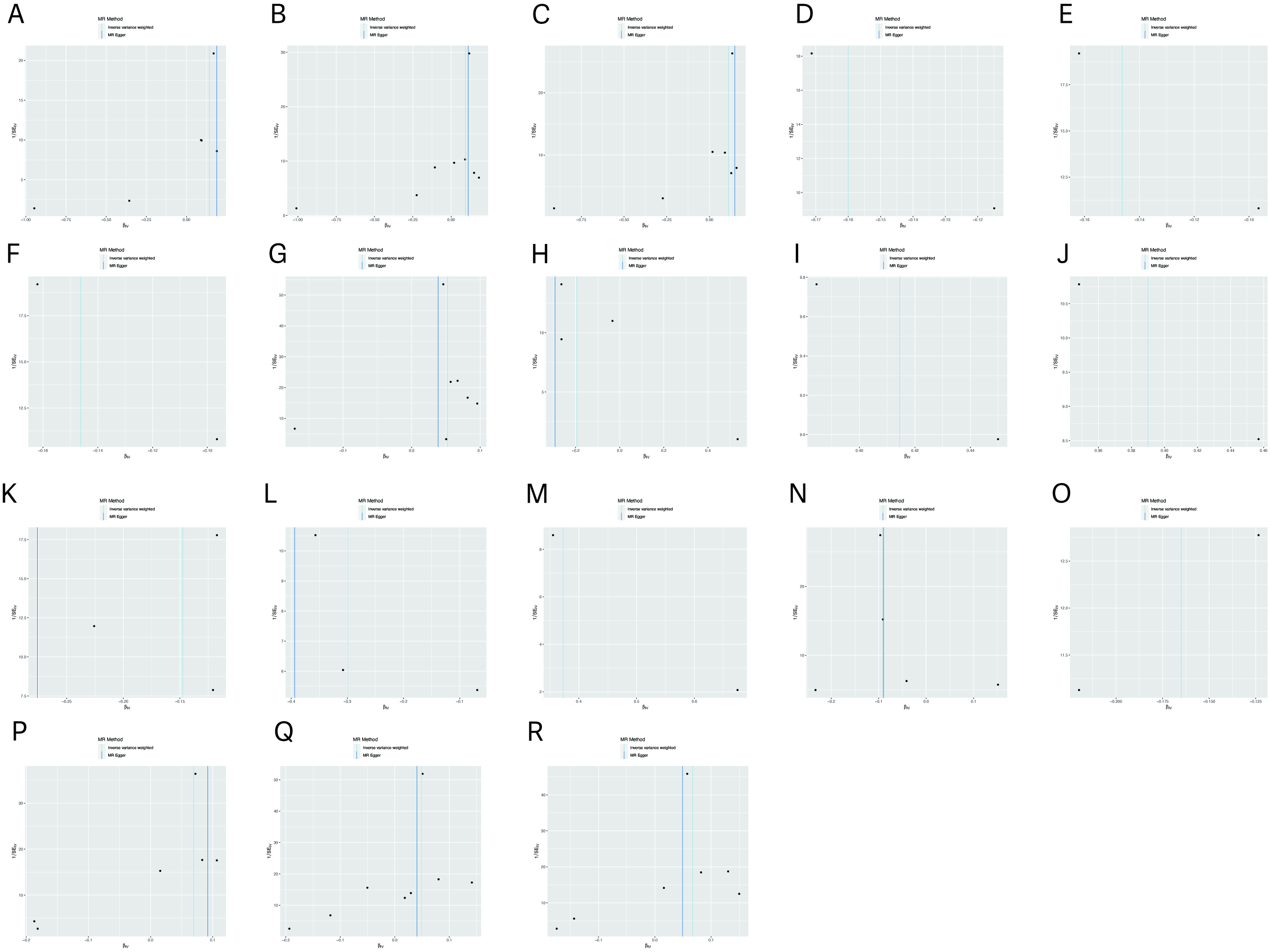

Supplement: Supplementary file 1 [file DataSheet1.zip › supplementary Material/FS3-HDP.tif]

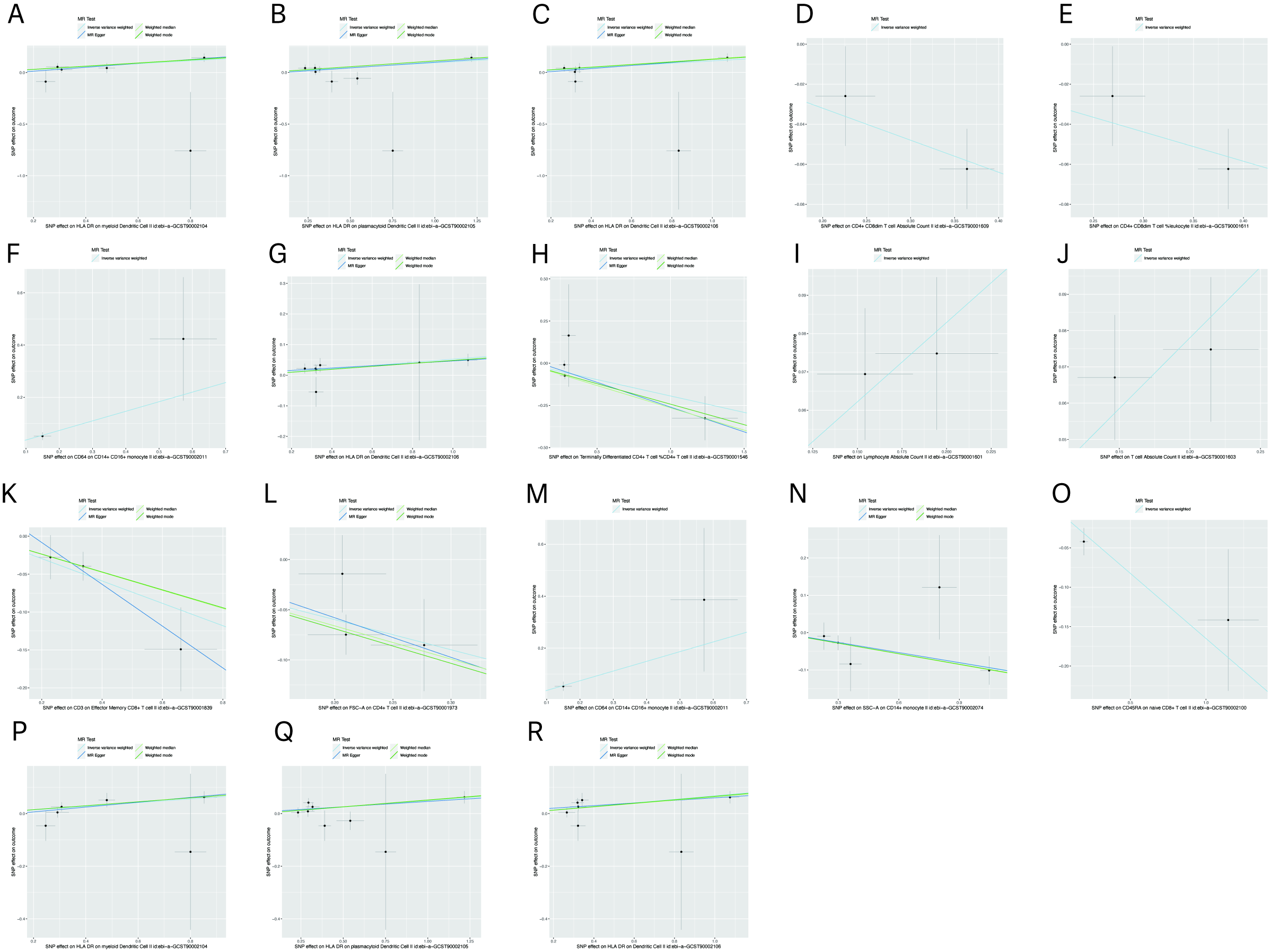

Supplement: Supplementary file 1 [file DataSheet1.zip › supplementary Material/FS1-HDP.tif]
